# A General Concurrent Template Strategy for Ordered Mesoporous Intermetallic Nanoparticles with Controllable Catalytic Performance

**DOI:** 10.1002/anie.202116179

**Published:** 2022-03-03

**Authors:** Hao Lv, Huaiyu Qin, Katsuhiko Ariga, Yusuke Yamauchi, Ben Liu

**Affiliations:** ^1^ Key Laboratory of Green Chemistry and Technology of Ministry of Education College of Chemistry Sichuan University Chengdu 610064 China; ^2^ JST-ERATO Yamauchi Materials Space-Tectonics Project International Research Centre for Materials Nanoarchitechtonics (WPI-MANA) National Institute for Materials Science (NIMS) 1-1 Namiki, Tsukuba Ibaraki 305-0044 Japan; ^3^ Australian Institute for Bioengineering and Nanotechnology (AIBN) School of Chemical Engineering The University of Queensland Brisbane QLD 4072 Australia

**Keywords:** Hydrogenation, Intermetallic Nanoparticles, Mesoporous Metals, Selective Catalysis, Template Synthesis

## Abstract

We report a general concurrent template strategy for precise synthesis of mesoporous Pt‐/Pd‐based intermetallic nanoparticles with desired morphology and ordered mesostructure. The concurrent template not only supplies a mesoporous metal seed for re‐crystallization growth of atomically ordered intermetallic phases with unique atomic stoichiometry but also provides a nanoconfinement environment for nanocasting synthesis of mesoporous nanoparticles with ordered mesostructure and rhombic dodecahedral morphology under elevated temperature. Using the selective hydrogenation of 3‐nitrophenylacetylene as a proof‐of‐concept catalytic reaction, mesoporous intermetallic PtSn nanoparticles exhibited remarkably controllable intermetallic phase‐dependent catalytic selectivity and excellent catalytic stability. This work provides a very powerful strategy for precise preparation of ordered mesoporous intermetallic nanocrystals for application in selective catalysis and fuel cell electrocatalysis.

## Graphical abstract

A general concurrent template strategy was reported for precise synthesis of mesoporous intermetallic nanoparticles with desired morphology, ordered mesostructure, and controlled intermetallic phases. The resultant mesoporous intermetallic PtSn nanoparticles exhibited the phase‐dependent selectivity and excellent stability for selective hydrogenation of 3‐nitrophenylacetylene.

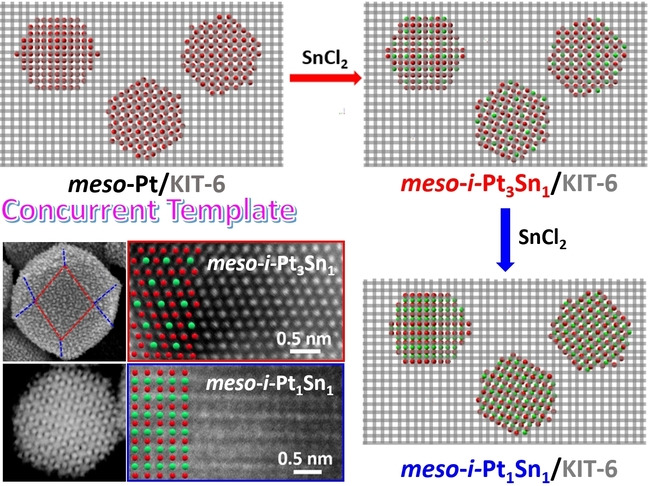

## Introduction

The past two decades have witnessed the rapid development of atomically ordered intermetallic nanoparticles for application in catalysis and electrocatalysis.^[1]^ Compared to traditional random alloys, the metal atoms in intermetallic nanoparticles are highly ordered, bonded by strong *d*‐orbital interactions and have well‐defined and unique atomic stoichiometries.^[2]^ These intrinsic features modify the surface geometric and electronic structures of intermetallic nanoparticles and thus increase their catalytic activity, selectivity, and stability.^[3]^ Thermal annealing is the most straightforward and commonly used method to prepare intermetallic nanoparticles, in which the stoichiometric arrangements and *d*–*d* orbital interactions of metal atoms were thermodynamically balanced and further engineered at elevated temperatures.[^2b^, ^4^] However, the high‐temperature annealing also causes severe atomic interdiffusion, leading to aggregation, making it difficult to control the morphology and structure of intermetallic nanomaterials.^[5]^

Mesoporous metal nanoparticles are a new subcategory of nanostructured materials in which the solid frameworks of metal nanocrystals are surrounded by 2–50 nm mesopores and built into an integral and uniform nanoparticle.^[6]^ As the second‐generation mesoporous materials, mesoporous metals have exhibited multiple structural advantages compared to their corresponding bulk and/or supported counterparts. First, high mesoporosity endows mesoporous metals with more catalytically active sites (higher surface areas, than same nanoparticles), which thus remarkably enlarges utilization efficiency of precious noble metals and boosts their catalytic mass activity. Second, continuous crystalline framework strongly accelerates transports of electrons and also inhibits physical Ostwald ripening processes (compared to discrete nanoparticles), which activates and stabilizes mesoporous metals accordingly.^[7]^ Third, concave/convex frameworks and confined environments modify surface electronic structures of mesoporous metal nanocrystals and provide “nanopincer” environments for reactants, especially key intermediates, which thus change the catalytic trends/barriers and possibly optimize catalytic selectivity toward desired products.^[8]^ However, it is still challenging to precisely synthesize mesoporous metals with controlled macroscopic morphology, ordered mesoscopic and atomic structure because the high‐surface‐energy metal nanocrystals migrate out the templates generally required to prepare mesoporous metals. To our knowledge, subtly combining periodically ordered mesoporous structure and atomically ordered intermetallic phase in one material, known as mesoporous intermetallics, has not been reported, even though they would theoretically perform well as catalysts.

In this work, we developed a general concurrent template strategy for precise synthesis of mesoporous intermetallic nanoparticles with well‐defined morphology, periodically ordered mesostructure, and atomically ordered crystalline phase. The synthesis started with mesoporous Pt nanocrystals confined within an ordered mesoporous sieve of KIT‐6 (defined as *meso*‐Pt/KIT‐6 hereafter), which was then utilized as a concurrent template to synthesize atomically ordered mesoporous intermetallic PtM nanoparticles. All of the evolutions from *meso*‐Pt to mesoporous intermetallic PtM (*meso*‐*i*‐PtM) nanoparticles happened in hard KIT‐6 template, thus stabilizing mesoporous stucture at elevated temperature. Six intermetallic phases and three mesoporous structures were synthesized as the examples, confirming the universality of the concurrent template strategy. Intermetallic crystalline phase‐dependent catalytic performance, with an assistance of crystalline mesoporosity, on the selective hydrogenation reaction of 3‐nitrophenylacetylene (3‐NPA) was finally evaluated, in whichmesoporous intermetallic PtSn showed controllable selectivity and high stability.

## Results and Discussion

**Figure 1:**
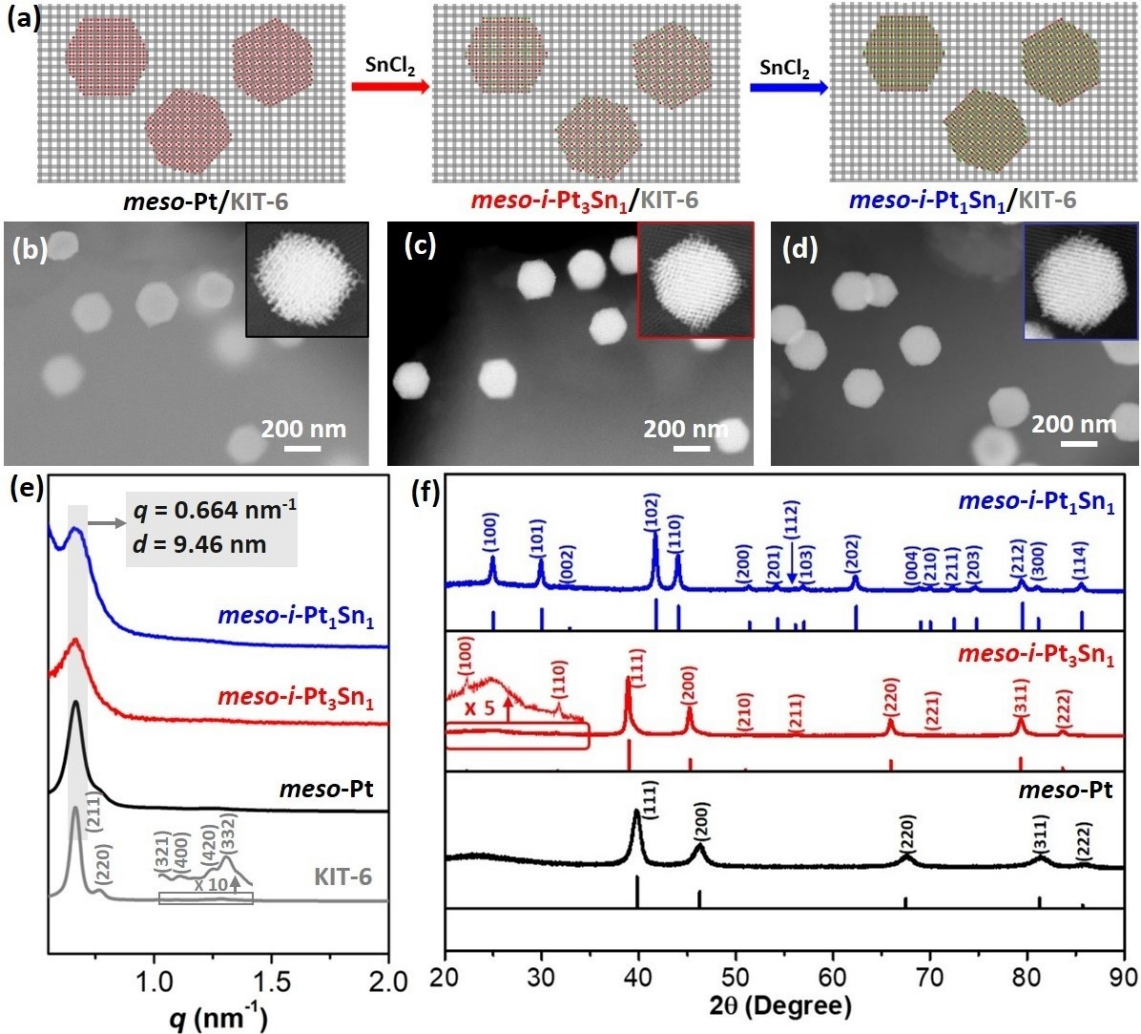
a) Synthesis of ordered *meso*‐*i*‐Pt_3_Sn_1_ and *meso*‐*i*‐Pt_1_Sn_1_ nanoparticles via a concurrent template strategy. HAADF‐STEM images of b) *meso*‐Pt/KIT‐6 template, c) *meso*‐*i*‐Pt_3_Sn_1_/KIT‐6 and d) *meso*‐*i*‐mesoPt_1_Sn_1_/KIT‐6 intermediates. Insets in b–d) are enlarged HAADF‐STEM images, indicating mesoporous Pt/Pt_3_Sn_1_/Pt_1_Sn_1_ confined in KIT‐6 template. e) SAXS and f) PXRD patterns of ordered *meso*‐Pt, *meso*‐*i*‐Pt_3_Sn_1_, and *meso*‐*i*‐Pt_1_Sn_1_ nanoparticles.

The concurrent template strategy to synthesize mesoporous intermetallic PtSn (*meso*‐*i*‐Pt_3_Sn_1_ and *meso*‐*i*‐Pt_1_Sn_1_) is schematically illustrated in Figure 1a (as the examples). A concurrent template of *meso*‐Pt/KIT‐6 was first synthesized by in‐situ reduction and nucleation growth of *meso*‐Pt nanocrystals within a highly ordered mesoporous KIT‐6 (see KIT‐6 in Figure S1a).^[7a]^ High‐angle annular dark‐field scanning transmission electron microscopy (HAADF‐STEM) image showed that *meso*‐Pt nanoparticles with an average diameter of 208 nm were homogeneously inserted in the KIT‐6 template (Figure 1b). Obviously, *meso*‐Pt was morphologically polyhedral, rather than spherical nanoparticles as commonly reported. Then, *meso*‐Pt/KIT‐6 hybrid as concurrent template was mixed with SnCl_2_ powder with different Pt/Sn ratios and directly annealed at 300 °C under a H_2_/N_2_ (5 : 95) atmosphere for different times (Table S1). During these steps, intermetallic PtSn nanocrystals did not aggregate and were still well dispersed in KIT‐6 template with no breakdown in polyhedral morphology or mesoporous structure (Figure 1c, d). The average diameter of *meso*‐*i*‐Pt_3_Sn_1_ and *meso*‐*i*‐Pt_1_Sn_1_ nanoparticles slightly increased to 224 and 243 nm, respectively, which was attributed to the gradual interruption of Sn into Pt nanocrystals (Figure S2).^[4a]^ Such an increasement in nanoparticles sizes was also confirmed by dynamic light scattering (DLS) (Figure S3). Besides, N_2_ sorption experiment confirmed that, with the interruption of Sn, surface areas of *meso*‐*i*‐PtSn/KIT‐6 hybrid and *meso*‐*i*‐PtSn also decreased slightly (Figure S4). Meanwhile, Pt dispersion of *meso*‐Pt was estimated as ≈20 %, which was slightly smaller than commercial Pt/C (38 %) but ≈37 times higher than the nanoparticles having a same diameter (208 nm) (≈0.54 %). Finally, mesoporous KIT‐6 and unreacted Sn precursors (or other impurities) were removed by washing with ethanol/H_2_O and etching with hydrofluoric acid (HF) to obtain ordered *meso*‐*i*‐Pt_3_Sn_1_ and *meso*‐*i*‐Pt_1_Sn_1_ nanoparticles (see Materials and Methods in Supporting Information for more details).

Various advanced characterization methods were used to confirm successful synthesis of ordered *meso*‐*i*‐Pt_3_Sn_1_ and *meso*‐*i*‐Pt_1_Sn_1_ nanoparticles via the concurrent template strategy. Small‐angle X‐ray scattering (SAXS) measurements showed that initial KIT‐6 template displayed a single set of peaks, corresponding to a double gyroid *Ia* $\bar{3}$ *d* mesostructure (Figure 1e). After the removal of KIT‐6, *meso*‐Pt, *meso*‐*i*‐Pt_3_Sn_1_ and *meso*‐*i*‐Pt_1_Sn_1_ nanoparticles retained the main signals. The *d*‐spacing of all the samples was around 9.5 nm, which matched well the periodicity of KIT‐6, indicating that they perfectly replicated the ordered mesostructure of KIT‐6. Powder X‐ray diffraction (PXRD) patterns were characterized to reveal the atomic crystalline phase structures (Figure 1f). Five typical diffraction peaks located at 35–90° confirmed the face‐centered‐cubic (*fcc*) crystal structure of monometallic *meso*‐Pt. Interestingly, *meso*‐*i*‐Pt_3_Sn_1_ and *meso*‐*i*‐Pt_1_Sn_1_ nanoparticles had completely different PXRD peaks, corresponding to atomically ordered intermetallic phases (rather than random alloys). Among them, *meso*‐*i*‐Pt_3_Sn_1_ was crystallographically Cu_3_Au‐type (*Pm* $\bar{3}$ *m* space group), while *meso*‐*i*‐Pt_1_Sn_1_ was NiAs‐type (*P*6_3_/*mmc* space group).^[3g]^

**Figure 2:**
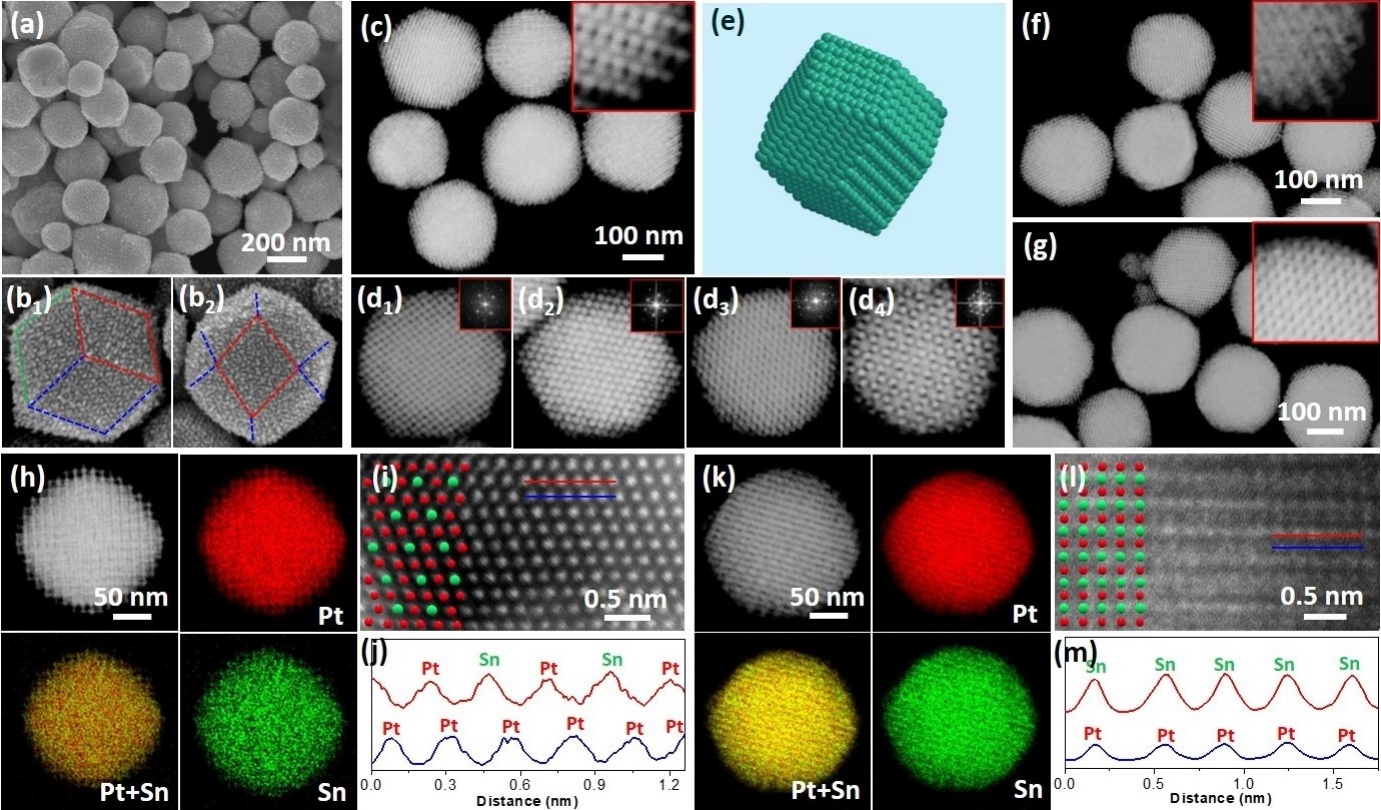
a) Low‐magnification and b) high‐magnification SEM images, c) low‐magnification and d) high‐magnification HAADF‐STEM images and corresponding FT patterns, and e) simulated scheme of *meso*‐*i*‐Pt_1_Sn_1_ nanoparticles. HAADF‐STEM images of f) *meso*‐Pt and g) *meso*‐*i*‐Pt_3_Sn_1_ nanoparticles. Insets in c, f, g) are corresponding enlarged HAADF‐STEM images. h) STEM EDX mappings, i) atomic‐resolution STEM image and j) corresponding intensity profiles of *meso*‐*i*‐Pt_3_Sn_1_ nanoparticles. k) STEM EDX mappings, l) atomic‐resolution STEM image and m) corresponding intensity profiles of *meso*‐*i*‐Pt_1_Sn_1_ nanoparticles.

The structure and morphology of *meso*‐*i*‐Pt_3_Sn_1_ and *meso*‐*i*‐Pt_1_Sn_1_ nanoparticles were further characterized by electron microscopy. Scanning electron microscopy (SEM) images showed that discrete nanoparticles were produced with good dispersity and homogeneity (Figure 2a). Surprisingly, most of the *meso*‐*i*‐Pt_1_Sn_1_ nanoparticles were polyhedral with a rhombic dodecahedral morphology of the nanocrystals. External surfaces of each nanoparticle were exactly bounded by twelve rhombic planes, confirming that they had a cubic *m* $\bar{3}$ *m* point‐group symmetry (Figure 2b). Both *meso*‐Pt and *meso*‐*i*‐Pt_3_Sn_1_ nanoparticles had the same rhombic dodecahedral morphology (Figure S5), indicating the morphology of mesoporous intermetallics was derived from the initial monometallic *meso*‐Pt. High‐magnification SEM images showed abundant nanowires replicated from the double gyroid KIT‐6 template, suggesting a highly ordered *Ia* $\bar{3}$ *d* mesostructure. Low‐magnification STEM images also showed that *meso*‐*i*‐Pt_1_Sn_1_ nanoparticles had a rhombic dodecahedral morphology (Figures 2c and S6). There were numerous ordered mesoporous frameworks within the nanoparticles. To investigate mesoporous structure, we further characterized individual nanoparticles along different mesoscopic directions. As evidenced by STEM images and corresponding Fourier transform (FT) patterns (Figure 2d), mesopores were periodically ordered and interconnected in the whole nanoparticles with an inverse double gyroid *Ia* $\bar{3}$ *d* mesostructure related to the initial KIT‐6, which built into macroscopic rhombic dodecahedra (Figure 2e). The framework thickness of mesoporous intermetallics wasin the range of 3.8–4.9 nm. Ordered and penetrated mesopores were further confirmed by tomography studies of a small nanoparticle (Videos S1 and S2). Similarly, *meso*‐Pt and *meso*‐*i*‐Pt_3_Sn_1_ nanoparticles were also macroscopically and mesoscopically ordered (Figure 2f, g), further confirming that mesoporous intermetallics were derived from the concurrent template of *meso*‐Pt/KIT‐6 intermediate. Meanwhile, electrochemical tests revealed that electrocatalytically active surface areas (ECSAs) are 26 m^2^ g_Pt_^−1^ for *meso*‐Pt, 28 m^2^ g_Pt_^−1^ for *meso*‐*i*‐Pt_3_Sn_1_, and 31 m^2^ g_Pt_^−1^ for *meso*‐*i*‐Pt_1_Sn_1_, respectively. By contrast, commercial Pt/C exhibited a larger ECSA of 56 m^2^ g_Pt_^−1^, mostly because of its smaller size of ≈3 nm (Figure S7).

Characterization of crystalline phases revealed atomically ordered intermetallic phases. STEM energy‐dispersive X‐ray (EDX) mapping showed that both Pt and Sn elements were uniformly distributed in the intermetallic nanoparticles with no phase‐separated compositions (Figure 2h, k). The molar ratios of *meso*‐*i*‐Pt_3_Sn_1_ and *meso*‐*i*‐Pt_1_Sn_1_ in the nanoparticles were estimated to be 75.3/24.7 and 50.1/49.9, respectively, perfectly consistent with the Pt_3_Sn_1_ and Pt_1_Sn_1_ intermetallic phases, which also agreed well with the data obtained from inductively coupled plasma‐mass spectrometry (ICP‐MS) (75.1/24.9 and 50.2/49.8) (Table S1). Furthermore, atomic‐resolution STEM image showed that *meso*‐*i*‐Pt_3_Sn_1_ had a Z‐contrast difference between Pt and Sn, corresponding to a L1_2_‐type phase structure (Figure 2i), which was also confirmed by the intensity profiles (Figure 2j). In contrast, a layer‐by‐layer atomic arrangement along the (001) plane was seen with *meso*‐*i*‐Pt_1_Sn_1_, implying a L1_0_‐type crystalline phase (Figure 2l, m). The morphology and structure characterizations demonstrated that we had successfully synthesized mesoporous intermetallic nanoparticles with macroscopically rhombic dodecahedral morphology, mesoscopically double gyroid *Ia* $\bar{3}$ *d* structure, and atomically ordered intermetallic crystalline phases.

The utilization of the *meso*‐Pt/KIT‐6 intermediate as the concurrent template is the key for the preparation of ordered mesoporous intermetallic nanoparticles. It had at least two important effects. First, *meso*‐Pt in the concurrent *meso*‐Pt/KIT‐6 can behave as the parent seed for subsequent in‐situ re‐crystallization growth of ordered intermetallic PtSn phases by inserting Sn into Pt nanocrystals. The synthesis of bimetallic intermetallic phases generally requires high temperature to increase the mobility and accelerate the reduction kinetics of two different metal precursors. The use of meso‐Pt/KIT‐6 can prevent the direct co‐crystallization of intermetallic nanoparticles. In a control experiment, direct reduction of PtCl_4_^2−^/Sn^2+^/KIT‐6 resulted in disordered nanoparticle aggregates (Figure S8). Second, KIT‐6 in concurrent *meso*‐Pt/KIT‐6 is not only utilized as the mesoporous nanocasting template for the formation of an ordered mesoporous structure but also provides the nanoconfinement environment to inhibit the overgrowth and migration of metal nanocrystals out of the KIT‐6 framework. When *meso*‐Pt was used as the sole template and KIT‐6 was mixed physically in the synthesis, the resulting PtSn intermetallic nanoparticles were solid without obvious mesoporous channels and there was a dramatic breakdown of morphology (Figure S9). Furthermore, since ordered *meso*‐*i*‐PtSn nanoparticles were inherited from *meso*‐Pt confined in KIT‐6, we could readily regulate their sizes in the range of 120 and 250 nm by changing the sizes of the *meso*‐Pt seeds in the *meso*‐Pt/KIT‐6 concurrent template (Figure S10). These results highlighted the ability of the concurrent template strategy to precisely synthesize ordered mesoporous intermetallic nanoparticles.

**Figure 3:**
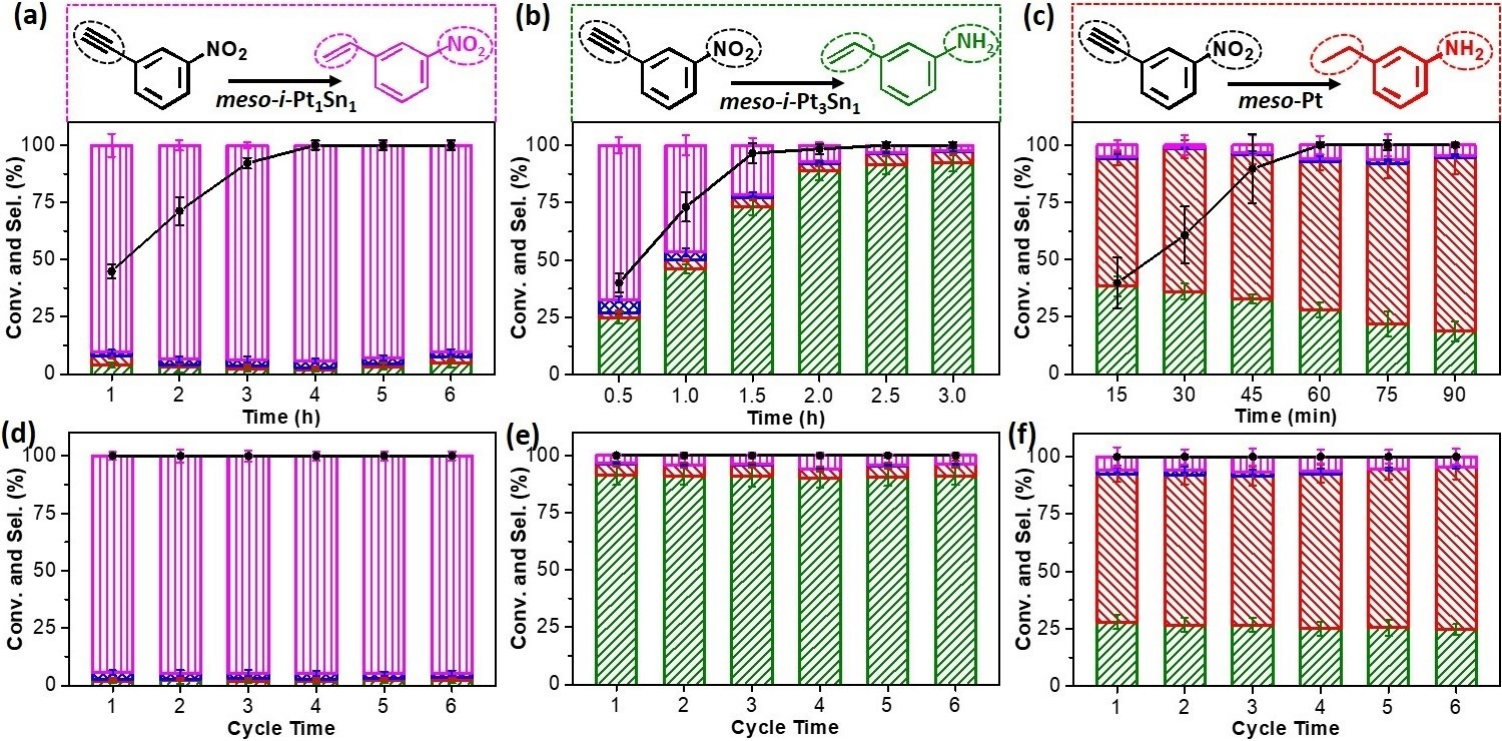
a–c) 3‐NPA conversion and product selectivity and d–f) catalytic cycling stability over a, d) *meso*‐*i*‐Pt_1_Sn_1_, b, e) *meso*‐*i*‐Pt_3_Sn_1_, and c, f) *meso*‐Pt nanoparticles. The reaction time for testing catalytic stability was 4 h for *meso*‐*i*‐Pt_1_Sn_1_, 2.5 h for *meso*‐*i*‐Pt_3_Sn_1_, and 1 h for *meso*‐Pt nanoparticles.

The catalytic performance of the *meso*‐*i*‐PtSn nanoparticles was evaluated on the selective hydrogenation reaction of 3‐NPA with ammonia borane as hydrogen source (Figure S11).^[9]^ Interestingly, our intermetallic nanoparticles demonstrated controllable selectivity towards three different reaction products depending on their atomic intermetallic phases. As shown in Figure 3a, *meso*‐*i*‐Pt_1_Sn_1_ converted 3‐NPA to the main product of 3‐nitrostyrene (3‐NS) (90.2 %) and trace byproducts (3‐nitroethylbenzene (3‐NE) (2.0 %), 3‐aminostyrene (3‐AS) (3.9 %), and 3‐aminoethylbenzene (3‐AE) (3.9 %)) after 1 h. The selectivity to 3‐NS increased to 95.1 % with a full 4 h conversion and was retained with longer catalytic times (6 h). By contrast, *meso*‐*i*‐Pt_3_Sn_1_ also favored the hydrogenation of 3‐NPA to 3‐NS (67.1 %) at an initial time (0.5 h), but quickly moved to the production of 3‐AS with a high selectivity of ≈91 % after full conversion of 3‐NPA (≈2 h) (Figure 3b). It should be highlighted that both NS and AS are industrially important intermediates for fine chemicals and petrochemicals.^[10]^ In contrast, monometallic *meso*‐Pt nearly over‐hydrogenated 3‐NPA to 3‐AE with a selectivity of ≈80 % when 3‐NPA was fully converted (Figure 3c), while the selectivity to 3‐AE slightly increased as the hydrogeneration reaction proceeded.

The surface electronic structures of ordered *meso*‐*i*‐PtSn nanoparticles, which corresponded strongly to their catalytic performance, were carried out thoroughly. First, X‐ray photoelectron spectroscopic (XPS) measurements showed that, with the formation of ordered intermetallics, the high‐resolution Pt 4f signal gradually shifted towards a higher binding energy, from 70.81 eV for *meso*‐Pt to 71.37 eV for *meso*‐*i*‐Pt_3_Sn_1_ and finally to 71.94 eV for *meso*‐*i*‐Pt_1_Sn_1_ (Figure S12a). Similarly, ordered *meso*‐*i*‐Pt_1_Sn_1_ and *meso*‐*i*‐Pt_3_Sn_1_ nanoparticles also negatively shifted the CO stripping potentials compared to *meso*‐Pt (0.17/0.15 V) (Figure S12b). These results confirmed that *meso*‐*i*‐Pt_1_Sn_1_ and *meso*‐*i*‐Pt_3_Sn_1_ had an electron‐deficiency Pt surface, which could change the adsorption/desorption features of reactive molecules and thus improve their catalytic selectivity.[^3d^, ^3g^, ^11^] Moreover, CO‐diffuse reflectance infrared Fourier transform spectroscopy (CO‐DRIFTS) exhibited different adsorption features of CO on catalysts (Figure S12c). For *meso*‐Pt, two strong adsorption peaks were seen, where the intense one corresponded to atop CO linearly adsorbed on a single Pt site (Pt−C=O) while the weak one was ascribed to CO adsorbed on the bridge Pt sites (Pt(Pt)=C=O). It confirmed a strong affinity of CO on monometallic *meso*‐Pt. In contrast, only one intense peak was observed for both *meso*‐*i*‐Pt_1_Sn_1_ and *meso*‐*i*‐Pt_3_Sn_1_, demonstrating different bridge Pt sites that possibly originated from the interruption of the staggered Sn atoms.[^4a^, ^12^] Meanwhile, the atop CO adsorption peaks of *meso*‐Pt negatively shifted towards the lower regions compared to *meso*‐*i*‐Pt_1_Sn_1_ and *meso*‐*i*‐Pt_3_Sn_1_. These results further confirmed different chemisorption properties of *meso*‐Pt, *meso*‐*i*‐Pt_1_Sn_1_ and *meso*‐*i*‐Pt_3_Sn_1_, which possibly corresponded to different catalytic selectivity toward different products.

Catalysts stability is another important parameter for their practical applications. Here, catalytic stability was performed by injecting a new proportion of reactants to the previous solution. Impressively, due to the highly ordered mesoporous structure and intermetallic phase, our materials exhibited excellent catalytic stability while maintaining their catalytic selectivity and activity even when the hydrogeneration reactions were repeated 6 times (Figure 3d–f). Advanced characterizations, including TEM and STEM EDS mapping images and PXRD patterns, confirmed the mesoscopic and atomic structures and elemental compositions after catalysis was the same as the fresh catalysts (Figures S13 and S14). The high selectivity and stability towards industrially important products suggested that our intermetallic catalysts have great potentials for practical applications in selective catalysis.

To further highlight the importance of mesoporous intermetallics, we further carried out catalytic tests of commercial Pt/C and Pt_1_Sn_1_@KIT‐6 (Pt_1_Sn_1_ nanoparticles embedded in mesoporous KIT‐6) for comparisons (Figure S15). As expected, Pt/C with ≈3 nm Pt nanoparticles fully hydrogenated 3‐NPA to 3‐AE (>95 %) within 3 h, indicating its high hydrogenation ability. In contrast, when catalyzed by Pt_1_Sn_1_@KIT‐6, ≈80 % of 3‐NS and ≈16 % of 3‐AS were achieved under the same conditions. Obviously, crystalline mesoporous framework slightly enhanced catalytic selectivity of intermetallic Pt_1_Sn_1_ toward favorable 3‐NS, possibly because crystalline mesoporosity provided a “pincer” environment for selective catalysis.^[8a]^ Meanwhile, both Pt and Pt_1_Sn_1_ nanoparticles gradually deactivated during catalysis, because of well‐known Ostwald ripening process. These results further confirmed that mesoporous structure not only stabilized the catalysts but also optimized catalytic selectivity partially.

**Figure 4:**
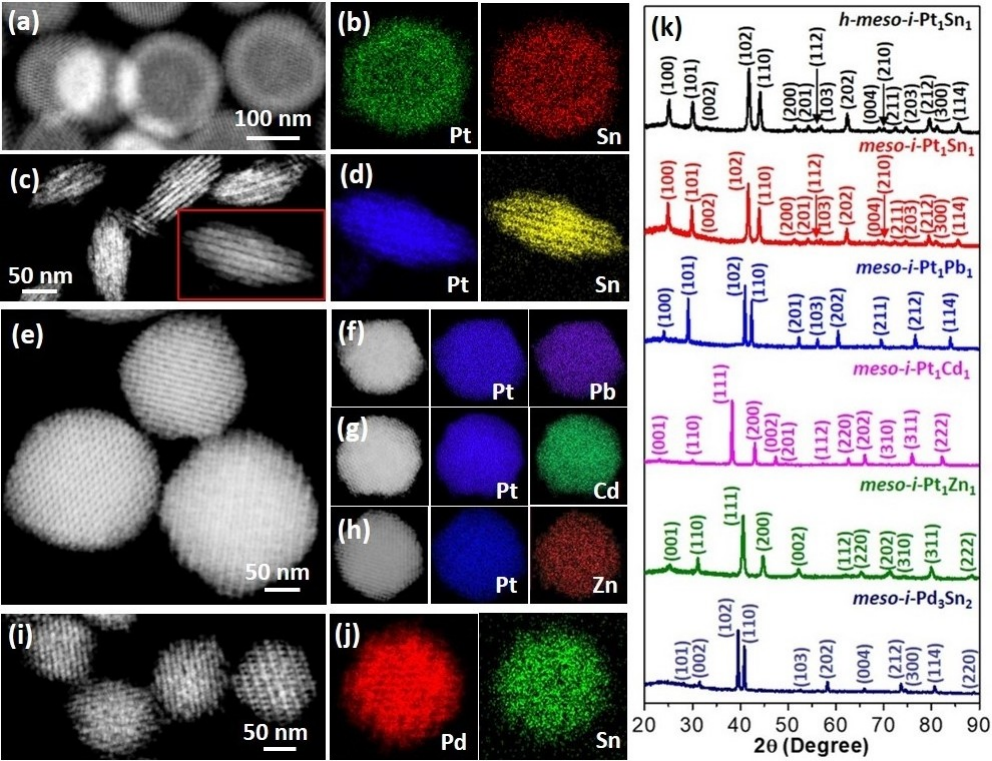
a) HAADF‐STEM image and b) EDX mappings of *h*‐*meso*‐*i*‐Pt_1_Sn_1_ nanoparticles. c) HAADF‐STEM image and d) EDX mappings of *meso*‐*i*‐Pt_1_Sn_1_ nanobundles. e) HAADF‐STEM image of *meso*‐*i*‐Pt_1_Pb_1_ nanoparticles. f–h) HAADF‐STEM images and corresponding EDX mappings of f) *meso*‐*i*‐Pt_1_Pb_1_, g) *meso*‐*i*‐Pt_1_Cd_1_, and h) *meso*‐*i*‐Pt_1_Zn_1_ nanoparticles. i) HAADF‐STEM image and j) EDX mappings of *meso*‐*i*‐Pd_3_Sn_2_ nanoparticles. k) PXRD patterns of *h*‐*meso*‐*i*‐Pt_1_Sn_1_ nanoparticles, *meso*‐*i*‐Pt_1_Sn_1_ nanobundles, *meso*‐*i*‐Pt_1_Pb_1_, *meso*‐*i*‐Pt_1_Cd_1_, *meso*‐*i*‐Pt_1_Zn_1_, and *meso*‐*i*‐Pd_3_Sn_2_ nanoparticles.

This concurrent template strategy is not only suitable for synthesizing *meso*‐*i*‐PtSn nanoparticles with controlled morphology and structure but also can be extended to other mesoporous intermetallics with different morphologies/structures and elemental compositions. We first engineered macroscopic morphologies and mesoporous structures with *meso*‐*i*‐Pt_1_Sn_1_. When being treated at a higher temperature (400 °C) and a higher gas flow rate (0.15 L min^−1^), the resultant products were ordered *meso*‐*i*‐Pt_1_Sn_1_ nanoparticles with a uniform interior hollow cavity (*h*‐*meso*‐*i*‐Pt_1_Sn_1_, Figure 4a, b). This was mostly because of the Kirkendall effect in which Pt atoms of *meso*‐Pt nanocrystals gradually diffused outward and further recrystallized with in‐diffusion Sn to form PtSn intermetallic nanoparticles on initial Pt nanoparticles. Meanwhile, confined KIT‐6 templated Kirkendall cavitation of hollow mesoporous PtSn intermetallics (see synthetic strategy in Figure S16).^[13]^ By contrast, when a *meso*‐Pt/SBA‐15 intermediate with a hexagonal mesostructure (see SBA‐15 in Figure S1b) was used as the concurrent template, *meso*‐*i*‐Pt_1_Sn_1_ with a rod‐like nanobundle morphology was obtained (Figure 4c, d, see synthetic strategy in Figure S17). Both nanoparticles had a L1_0_‐type Pt_1_Sn_1_ intermetallic phase, as demonstrated by PXRD patterns (the top two patterns in Figure 4k).

Three other *meso*‐*i*‐Pt_1_M_1_ nanoparticles with rhombic dodecahedral morphology, ordered *Ia* $\bar{3}$ *d* mesostructure and controlled intermetallic phase were also prepared using the *meso*‐Pt/KIT‐6 intermediate as concurrent template and corresponding metal nitrates as metal sources. 5*d* metal of Pb, 4*d* metal of Cd, and 3*d* metal of Zn were successfully alloyed within Pt nanocrystals and formed atomically ordered intermetallic nanoparticles (Figures 4e–h and S18). PXRD patterns suggested that they were *meso*‐*i*‐Pt_1_Pb_1_ (*P*6_3_/*mmc* space group), *meso*‐*i*‐Pt_1_Cd_1_ (*P*4/*mmm* space group), and *meso*‐*i*‐Pt_1_Zn_1_ (*P*4/*mmm* space group), respectively, with atomically ordered stoichiometries (Patterns 3–5 in Figure 4k). Moreover, Pd‐based intermetallic nanoparticles, for example *meso*‐*i*‐Pd_3_Sn_2_, were also prepared using *meso*‐Pd/KIT‐6 intermediate as the concurrent template under the same synthetic conditions (Figure 4i, j), since the monoclinic Pd_3_Sn_2_ phase was thermodynamically favorable (*P*6_3_/*mmc* space group, the bottom pattern in Figure 4k).^[14]^ The slightly smaller size of the nanoparticles was due to the quicker reduction rate of Pd precursors during the synthesis of *meso*‐Pd/KIT‐6 intermediate.^[15]^

## Conclusion

Using a *meso*‐Pt/KIT‐6 intermediate, wesuccessfully developed a general concurrent template strategy for precisely synthesizing mesoporous intermetallic nanoparticles with macroscopically rhombic dodecahedral morphology, mesoscopically ordered double gyroid *Ia* $\bar{3}$ *d* structure, and atomically ordered Pt_3_Sn_1_ and Pt_1_Sn_1_ intermetallic phases. The *meso*‐Pt nanocrystals in the concurrent template re‐crystallized with metal precursors (for example SnCl_2_) under the elevated temperatures to form atomically ordered intermetallics, while mesoporous silica (KIT‐6 or SBA‐15) provided a nanoconfinement mesoporous environment to grow ordered mesoporous nanocrystals. More importantly, the concurrent template method was readily extended to engineer macroscopic morphology, mesoscopic structure, and atomic intermetallic phase (orderliness) of other mesoporous intermetallic nanoparticles. These nanoparticles had optimum surface electronic states and chemisorption properties, with a controllable catalytic selectivity and excellent catalytic stability. Catalytic studies in selective hydrogeneration of 3‐NPA revealed that *meso*‐*i*‐Pt_1_Sn_1_, *meso*‐*i*‐Pt_3_Sn_1_, and *meso*‐Pt hold a controllable selective tendency toward 3‐NS, 3‐AS, and 3‐AE, respectively. This general method using *meso*‐Pt(Pd)/KIT‐6(SBA‐15) as the concurrent template opens up new opportunities for precise preparation of mesoporous intermetallic nanoparticles with well‐defined morphology, structure, and phase, which can be widely utilized as efficient heterogeneous catalysts for catalytic applications.

## Author Contributions

B. Liu directed the project. H. Lv, Y. Yamauchi, and B. Liu conceived the project and designed the experiments. H. Lv carried out the experiments, characterizations, and catalysis tests. H. Qin assisted the catalysis tests. K. Ariga commented the project. All of the authors discussed the experiments and co‐wrote the manuscript.

## Conflict of interest

The authors declare no conflict of interest.

1

## Supporting information

As a service to our authors and readers, this journal provides supporting information supplied by the authors. Such materials are peer reviewed and may be re‐organized for online delivery, but are not copy‐edited or typeset. Technical support issues arising from supporting information (other than missing files) should be addressed to the authors.

Supporting Information

Supporting Information

Supporting Information

## Data Availability

The data that support the findings of this study are available from the corresponding author upon reasonable request.
