# Supplementary material for: A General Concurrent Template Strategy for Ordered Mesoporous Intermetallic Nanoparticles with Controllable Catalytic Performance
Source: Angew Chem Int Ed Engl. 2022 Mar 3;61(17):e202116179. doi: 10.1002/anie.202116179 (PMC9311168; doi:10.1002/anie.202116179)
Supplement: Supplementary file 1 — Supporting Information [file ANIE-61-0-s002.pdf]

Angewandte Chemie
Eine Zeitschrift der Gesellschaft Deutscher Chemiker

Supporting Information

A General Concurrent Template Strategy for Ordered Mesoporous Intermetallic Nanoparticles with Controllable Catalytic Performance

H. Lv, H. Qin, K. Ariga, Y. Yamauchi, B. Liu**

Supporting Figures and Table Contents

Figure S1. Structural characterization of mesoporous KIT-6 and SBA-15.

Figure S2. Size distributions of *meso*-Pt, *meso*-*i*-Pt_3Sn_1, and *meso*-*i*-Pt_1Sn_1 nanoparticles.

Figure S3. DLS data of *meso*-Pt, *meso*-*i*-Pt_3Sn_1, and *meso*-*i*-Pt_1Sn_1 nanoparticles.

Figure S4. N_2 sorption data of *meso*-Pt, *meso*-*i*-Pt_3Sn_1, and *meso*-*i*-Pt_1Sn_1 nanoparticles.

Figure S5. SEM images of *meso*-Pt and *meso*-*i*-Pt_3Sn_1 nanoparticles.

Figure S6. Characterizations of *meso*-*i*-Pt_1Sn_1 nanoparticles by STEM, TEM and SEM.

Figure S7. Electrochemical active areas of *meso*-Pt, *meso*-*i*-Pt_3Sn_1, and *meso*-*i*-Pt_1Sn_1 nanoparticles.

Figure S8. *i*-PtSn nanoparticles obtained from $PtCl_4^{2-}/Sn^{2+}$/KIT-6.

Figure S9. *i*-PtSn nanoparticles obtained from *meso*-Pt/$SnCl_2$ and *meso*-Pt/$SnCl_2$/KIT-6.

Figure S10. Characterization of *meso*-*i*-Pt_1Sn_1 nanoparticles with different sizes.

Figure S11. Possible hydrogenation routes and products of 3-NPA.

Figure S12. Surface chemical features of *meso*-Pt, *meso*-*i*-Pt_3Sn_1, and *meso*-*i*-Pt_1Sn_1 nanoparticles.

Figure S13. Electron microscopy characterizations of samples after catalysis.

Figure S14. PXRD characterizations of samples after catalysis.

Figure S15. Catalytic performance of commercial Pt/C and *i*-Pt_1Sn_1@KOT-6.

Figure S16. Synthetic strategy of ordered *h*-*meso*-*i*-Pt_1Sn_1 nanoparticles.

Figure S17. Synthetic strategy of ordered *meso*-*i*-Pt_1Sn_1 nanobundles.

Figure S18. Characterization of *meso*-*i*-Pt_1Cd_1 and *meso*-*i*-Pt_1Zn_1 nanoparticles.

Table S1. Pt/Sn ratios during the synthesis of *meso*-*i*-Pt_3Sn_1 and *meso*-*i*-Pt_1Sn_1 nanoparticles.

Materials and Methods

Materials and Chemicals

Pluronic P123 (PEO_{20}-PPO_{70}-PEO_{20}, Purity 99 %), commercial Pt/C (20 wt % of Pt), 3-nitrophenylacetylene, and ammonia borane (AB) were obtained from Sigma-Aldrich. Potassium tetrachloroplatinate (K_2PtCl_4), sodium tetrachloropalladate (Na_2PdCl_4), lead nitrate ($Pb(NO_3)_2$), cadmium nitrate ($Cd(NO_3)_2$), zinc nitrate ($Zn(NO_3)_2$), tin(II) chloride ($SnCl_2$), hydrofluoric acid (HF), hydrochloric acid (HCl), sodium hydroxide (NaOH), ammonia solution (NH_3•H_2O, 50%), L-ascorbic acid (AA), 1-butanol, ethanol, and tetraethoxysilane (TEOS) were purchased from Sinopharm Chemical Reagent Co. Ltd. (Shanghai).

Synthesis of mesoporous KIT-6 and SBA-15

Mesoporous KIT-6 was synthesized according to previously reported procedures (*Chem. Commun.* **2003**, 2136; *Chem. Commun.* **2010**, *46*, 6365), while mesoporous SBA-15 was produced according to the reported literatures (*Science* **1998**, *279*, 548; *Angew. Chem. Int. Ed.* **2015**, *54*, 7060).

Synthesis of the concurrent template

The *meso*-Pt/KIT-6 concurrent template was synthesized by a classic nanocasting method as reported previously (*J. Am. Chem. Soc.* **2011**, *133*, 14526). In a typical synthesis, 0.30 g of KIT-6 and 80.0 mg of K_2PtCl_4 were dispersed in 2.0 mL of water, which was dried under vacuum to obtain a powder. Then, 2.0 mL of freshly prepared L-ascorbic acid (AA) solution (0.25 M) was added dropwise to the powder to start the crystallization of *meso*-Pt within mesoporous KIT-6. The reaction proceeded at room temperature for 10 h. After that, monometallic *meso*-Pt nanoparticles were obtained by etching with 10 % HF and washing three times with water/ethanol, and drying at 50 ^{o}C. The ***meso*-Pt/SBA-15** template was synthesized with the same procedure using SBA-15 as the initial template, while the ***meso*-Pd/KIT-6** template was obtained using Na_2PdCl_4 as the metal precursor.

Synthesis of *meso-i*-PtM intermetallic nanoparticles

The *meso-i*-PtM intermetallic nanoparticles were synthesized by a concurrent template strategy. In a typical synthesis for ***meso-i*-Pt_3Sn_1**, 0.20 g of as-synthesized *meso*-Pt/KIT-6 intermediate was thoroughly mixed with 5.0 mg of $SnCl_2$. Then, the mixture was treated at 300 ^{o}C for 4 h in a tube furnace under a H_2/N_2 atmosphere (5:95) with a flow rate of 0.05 L min^{-1}. Finally, *meso-i*-Pt_3M_1 nanoparticles were obtained by washing three times with HF and water/ethanol, and drying at 50 ^{o}C before use. The ***meso-i*-Pt_1Sn_1** was synthesized with the same procedures but using 25.0 mg of $SnCl_2$ as the Sn precursor and heat-treated for 6 h. The ***h-meso-i*-Pt_1Sn_1** nanoparticles were synthesized with the same procedures but using the higher flow rate (0.15 L min^{-1}) and calcination temperature (400 ^{o}C). Similarly, ***meso-i*-Pt_1Pb_1** was synthesized by treating the powder mixture

containing 0.20 g of *meso*-Pt/KIT-6 intermediate and 25.0 mg of $Pb(NO_3)_2$ at 300 ^oC for 5 h. The ***meso-i*-Pt_1Cd_1** was synthesized by treating the powder mixture containing 0.20 g of *meso*-Pt/KIT-6 and 25.0 mg of $Cd(NO_3)_2$ at 500 ^oC for 6 h. The ***meso-i*-Pt_1Zn_1** was synthesized by treating the powder mixture containing 0.20 g of *meso*-Pt/KIT-6 and 25.0 mg of $Zn(NO_3)_2$ at 500 ^oC for 6 h.

For ***meso-i*-Pd_3Sn_2**, 0.20 g of as-synthesized *meso*-Pd/KIT-6 intermediate was mixed with 25.0 mg of $SnCl_2$, and treated at 300 ^oC for 4 h in a tube furnace under the H_2/N_2 atmosphere (5:95) with a flow rate of 0.05 L min^{-1}. The *meso-i*-Pd_3Sn_2 nanoparticles were obtained by washing three times with HF and water/ethanol, and drying at 50 ^oC.

For **Pt_1Sn_1@KIT-6,** 0.30 g of KIT-6 and 80.0 mg of K_2PtCl_4 were dispersed in 10.0 mL of water. Then, 2.0 mL of freshly prepared, cold $NaBH_4$ solution (0.25 M) was injected to start the crystallization of Pt nanoparticles within mesoporous KIT-6 (Pt@KIT-6). The reaction proceeded at room temperature for 2 h. After that, Pt@KIT-6 was collected by centrifugating and washing three times with water/ethanol, and drying at 50 ^oC. Then, 0.20 g of as-synthesized Pt@KIT-6 was thoroughly mixed with 25.0 mg of $SnCl_2$, and further treated at 300 ^oC for 4 h in a tube furnace under a H_2/N_2 atmosphere (5:95) with a flow rate of 0.05 L min^{-1}. Pt_1Sn_1@KIT-6 was finally collected by washing three times with water/ethanol and drying at 50 ^oC before use.

Electrochemical experiments

Electrochemical cyclic voltammetry (CV) curves were performed on the CHI 660E electrochemical analyzer at 25 ^oC as reported in our previous works (*Sci. Bull.* **2020**, *65*, 1823; *Nano Lett.* **2019**, *19*, 3379). A three-electrodes system was used for all electrochemical tests, in which glassy carbon electrode (GCE, 0.07065 cm^2) was used as the working electrode, a carbon rod as the counter electrode, and a saturated calomel electrode as the reference electrode. An ink of the catalysts was prepared by mixing 0.5 mg of nanocatalysts, 0.5 mg of Vulcan XC-72 carbon, 0.4 mL of ethanol and 0.1 mL of H_2O. After sonicating for 0.5 h, 25 μL of Nafion solution (5 wt.% in alcohol and H_2O) was added and further sonicated for 0.5 h. Then, 6 μL of above-prepared ink solution was dropped on the GCE electrode and dried at 40 ^oC before test. The electrolyte solution was initially purged with N_2 for 30 min to remove O_2 and other gas before test.

For electrochemical CO stripping tests, the samples were first saturated with CO by bubbling the gas at 0.15 V in 0.5 M H_2SO_4 solution for 20 min. Excess CO was then eliminated from the electrochemical cell by purging the solution with pure N_2 for 30 min. Afterwards, four cyclic voltammograms were recorded at 0.05 V s^{-1} in a potential range of -0.2– 0.9 V (vs SCE). The first cycle provided the total charge of CO oxidation, while the other cyles were done to check the recovery of the original voltametric profile.

Selective hydrogenation reaction of 3-nitrophenylacetylene (3-NPA)

The selective hydrogenation of 3-NPA was performed in a 15 mL pressure bottle with 0.25 mL (1.0 mg mL^{-1} in ethanol solution) of catalyst, 10 mg of 3-NPA, 3.0 mg of AB, 0.025 mL of distilled water, and 0.70 mL of ethanol. The reaction was performed at 50 °C under magnetic stirring for different times. The products were analyzed by gas chromatography (GC) with a Panna A91 Plus GC equipped with a flame ionization detector and a DB-WAX capillary column (J&W, 30 m, 0.25 mm i.d.) with nitrogen as the carrier gas. For stability tests, the catalysts were recleaned and dried after catalysis. Meanwhile, the structure and composition of the catalysts after catalysis were characterized by TEM and STEM. The reaction time for catalytic stability tests was 4 h for *meso*-*i*-Pt_1Sn_1, 2.5 h for *meso*-*i*-Pt_3Sn_1, and 1 h for *meso*-Pt nanoparticles.

Characterization

SEM images were collected using a JEOL JSM-7600F field emission Scanning Electron Microscope. SEM samples were prepared by dropcasting a suspension of the sample powder onto a silicon wafer. TEM and STEM studies were carried out using a field emission TEM (JEM-F200, JEOL Ltd., Japan) with an accelerating voltage of 200 kV. TEM and STEM samples were prepared by dropcasting a diluted suspension of the sample powder onto a carbon coated copper grid (300 mesh). SAXS patterns were measured using an Anton Paar SAXSess mc^2 instrument. PXRD patterns of powder samples were recorded using a D/max 2500 VL/PC diffractometer (Japan) equipped with graphite-monochromatized Cu Kα radiation in 2θ ranging from 20° to 90°. The working voltage and current were 40 kV and 100 mA, respectively. BET surface area of catalysts was measured using an ASAP 2020 PLUS automated N_2 gas adsorption system. Before characterization, 50 mg of sample was degassed at 150 °C for 12 h to remove water and other physically adsorbed species. DLS was performed using Zetasizer Nano ZS90. XPS spectra were performed on a scanning X-ray microprobe (Thermo ESCALAB 250Xi) that uses Al Kα radiation. The binding energy of the C 1s peak (284.8 eV) was used as a standard to calibrate the binding energies of other elements (Pt and Sn). ICP-MS was recorded on a NexION 350D.

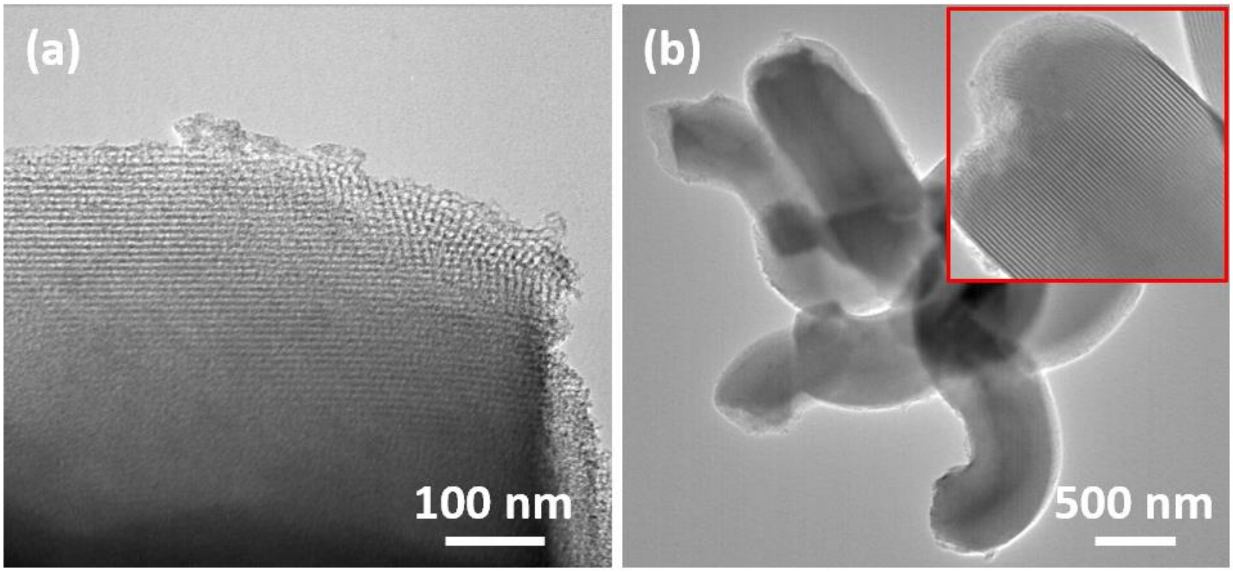

Figure S1. TEM images of (a) KIT-6 and (b) SBA-15 templates. KIT-6 has a double gyroid $Ia\bar{3}d$ mesostructure, while SBA-15 has a two-dimensional hexagonal *p*6*mm* mesostructure.

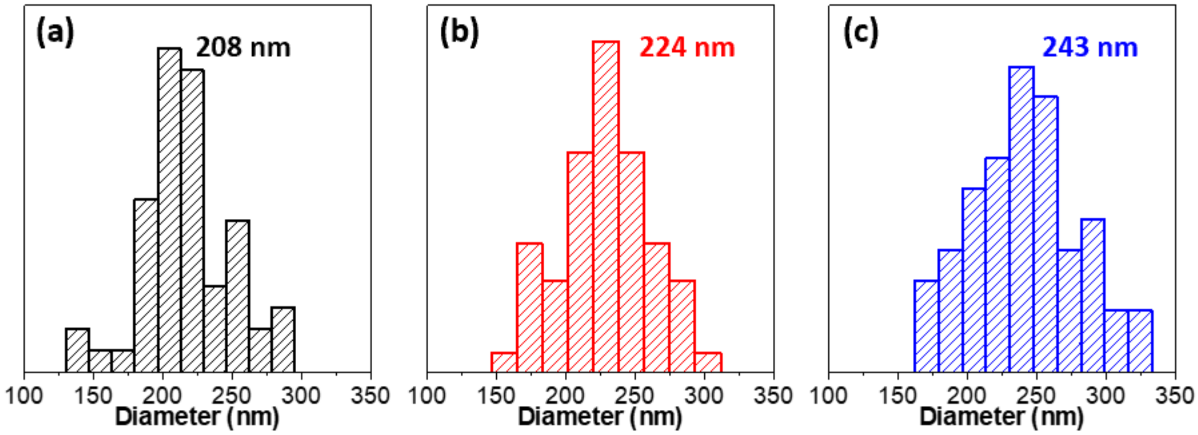

Figure S2. Size distributions of (a) *meso*-Pt, (b) *meso*-*i*-Pt_3Sn_1, and (c) *meso*-*i*-Pt_1Sn_1 nanoparticles. Obviously, with the insertion of Sn into Pt nanocrystals, the size of mesoporous intermetallic nanoparticles is slightly increased to 224 nm for *meso*-*i*-Pt_3Sn_1 and 243 nm for *meso*-*i*-Pt_1Sn_1.

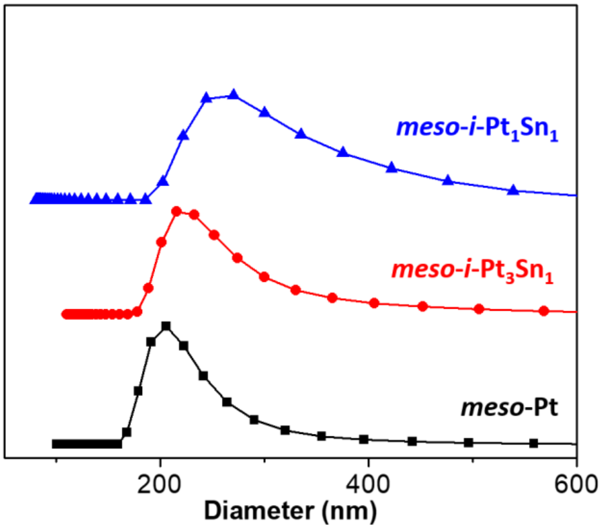

Figure S3. DLS profiles of *meso*-Pt, *meso*-*i*-Pt_3Sn_1, and *meso*-*i*-Pt_1Sn_1 nanoparticles, indicating a gradual increasement of particle sizes with insertion of Sn into *meso*-Pt.

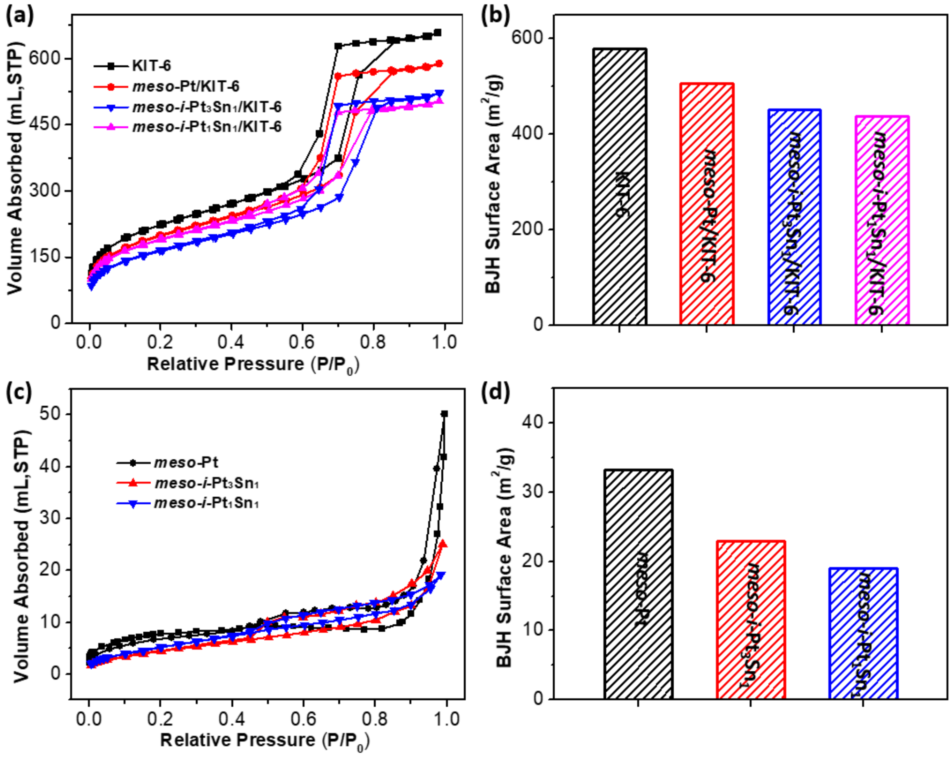

Figure S4. (a) N_2 sorption isotherms and (b) corresponding surface areas of KIT-6, *meso*-Pt/KIT-6, *meso*-*i*-Pt_3Sn_1/KIT-6, and *meso*-*i*-Pt_1Sn_1/KIT-6. (c) N_2 sorption isotherms and (d) corresponding surface areas of *meso*-Pt, *meso*-*i*-Pt_3Sn_1, and *meso*-*i*-Pt_1Sn_1.

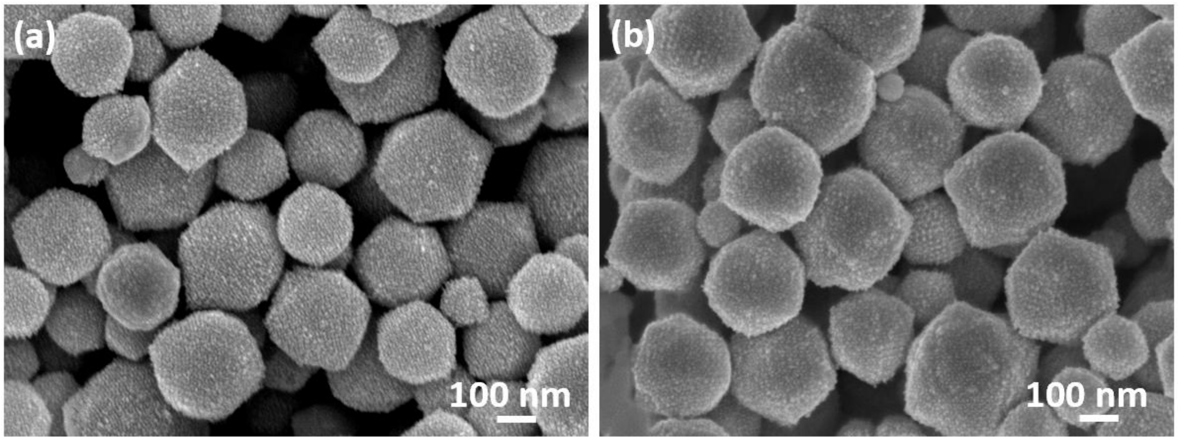

Figure S5. SEM images of (a) *meso*-Pt and (b) *meso*-*i*-Pt_3Sn_1 nanoparticles, confirming a rhombic dodecahedral morphology and ordered mesostructure. The morphology and structure of *meso*-Pt is almost same as *meso*-*i*-Pt_3Sn_1 and *meso*-*i*-Pt_1Sn_1, indicating they were derived from the concurrent template, *meso*-Pt/KIT-6.

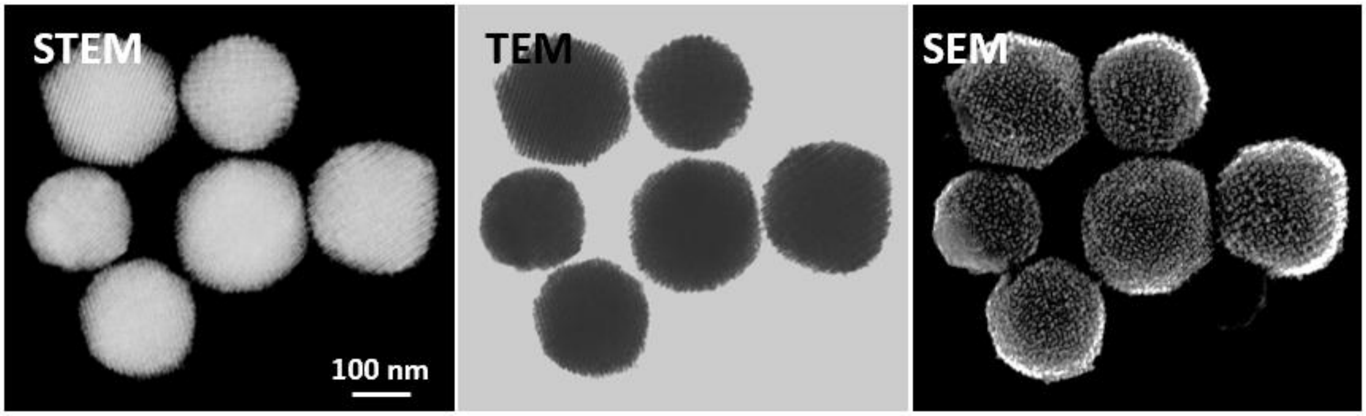

Figure S6. Structural characterization of *meso*-*i*-Pt_1Sn_1 nanoparticles by STEM, TEM, and SEM. Although the STEM image shows some disordered nanoparticles; it did not indicate that the mesostructure is disordered. In fact, the disordered appearance was originated from the different observation angles.

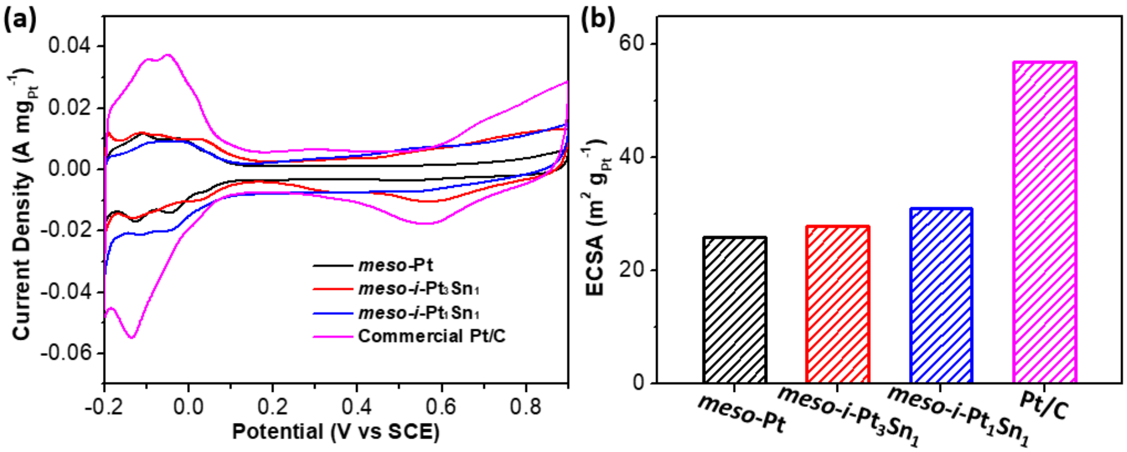

Figure S7. (a) Electrochemical CV curves and (b) corresponding ECSAs (normalized to Pt) of *meso*-Pt, *meso*-*i*-Pt_3Sn_1, and *meso*-*i*-Pt_1Sn_1, and commercial Pt/C.

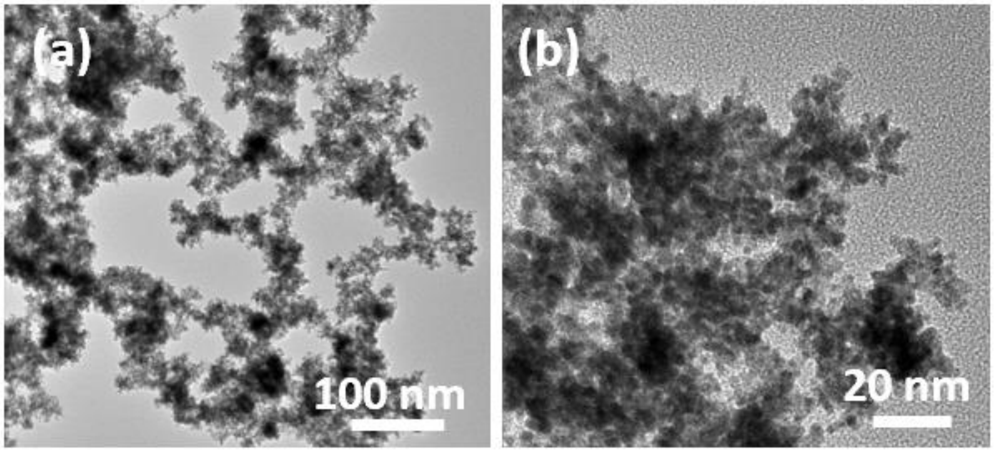

Figure S8. TEM images of PtSn nanoparticles synthesized by H_2/N_2 reduction of H_2PtCl_4, $SnCl_2$ and KIT-6. Due to the different reduction kinetics and mobility of metal precursors, the PtSn nanoparticles almost migrated out the KIT-6 framework, resulting in the formation of nanoparticles and their aggregates (rather than mesoporous nanoparticles).

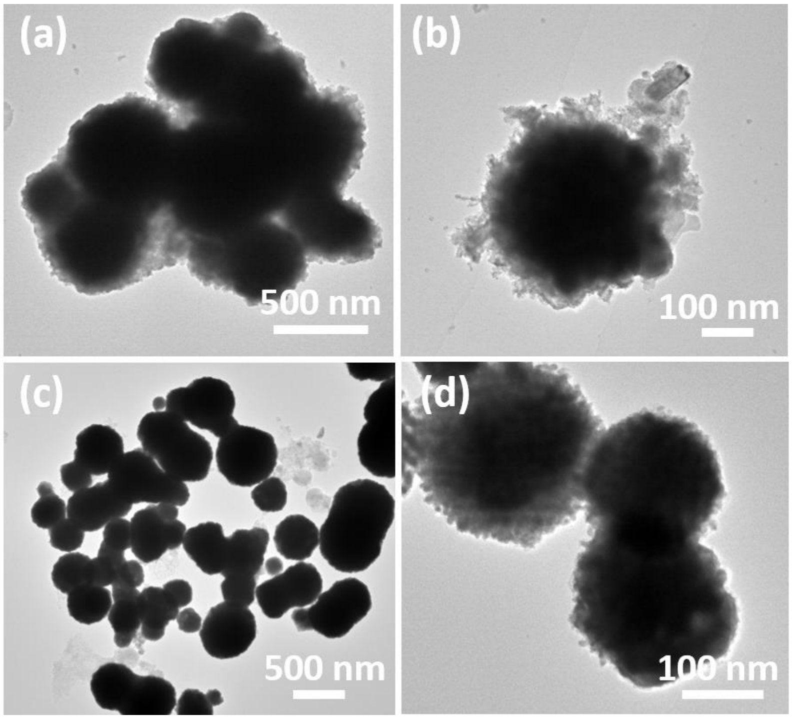

Figure S9. TEM images of PtSn nanoparticles synthesized by H_2/N_2 reduction of (a,b) *meso*-Pt and $SnCl_2$, and (c,d) *meso*-Pt, $SnCl_2$ and KIT-6 (*meso*-Pt was not confined in KIT-6). In the absence of a mesoporous nanoconfinement effect, the synthesis only produced larger nanoparticles without mesoporous channels.

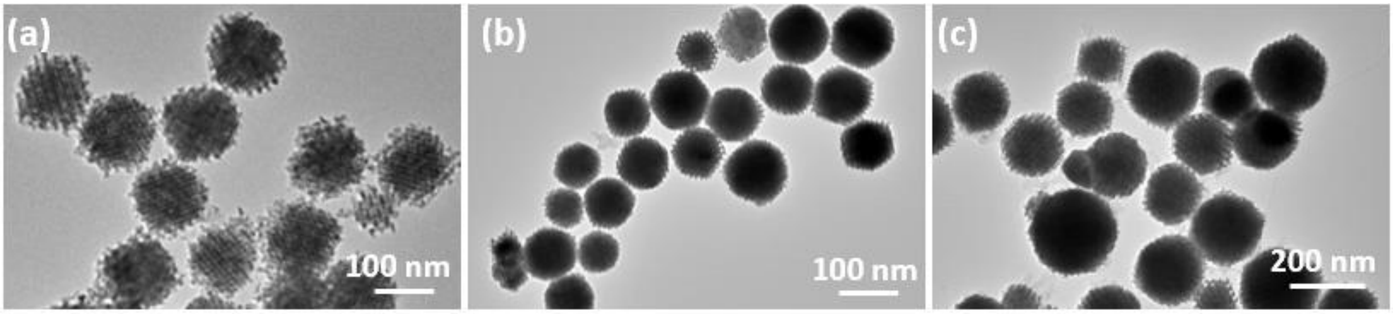

Figure S10. TEM images of *meso*-*i*-Pt_1Sn_1 nanoparticles with a nanoparticle size of (a) 121 nm, (b) 164 nm, and (c) 208 nm. By tuning the *meso*-Pt size in the *meso*-Pt/KIT-6 intermediate, the size of *meso*-*i*-Pt_1Sn_1 nanoparticles can be easily changed in the range of 120-250 nm.

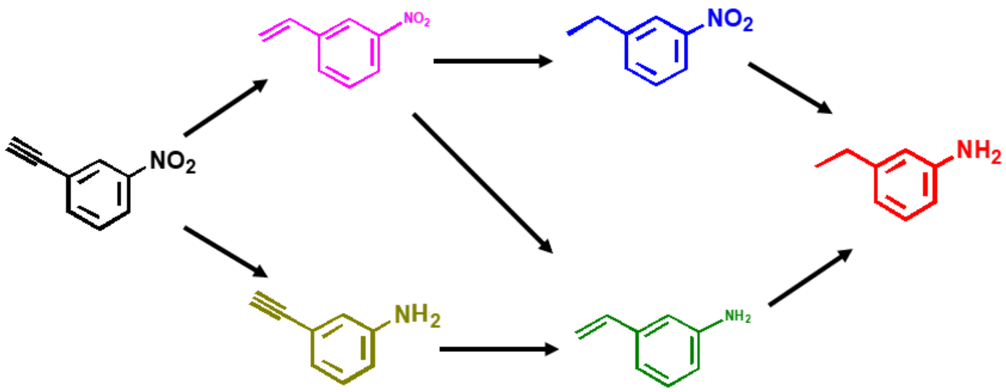

Figure S11. Possible hydrogenation routes and products of 3-NPA. There are two different routes for the hydrogenation reaction. One is the hydrogenation of nitryl group first, while the other is the hydrogenation of alkynyl (vinyl) groups first.

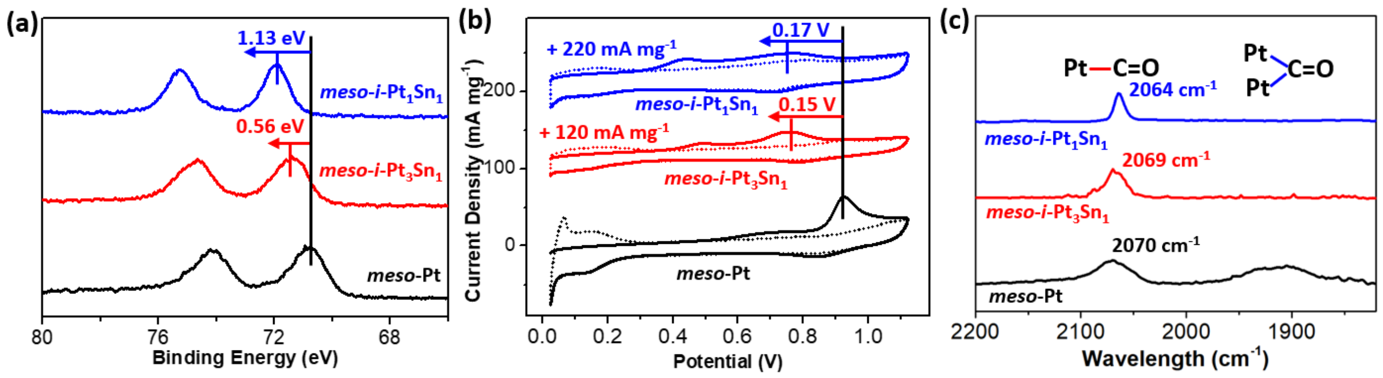

Figure S12. (a) High-resolution XPS Pt 4f spectra, (b) electrochemical CO stripping voltammetry, and CO DRIFTS spectra of *meso*-*i*-Pt_1Sn_1, *meso*-*i*-Pt_3Sn_1, and *meso*-Pt nanoparticles.

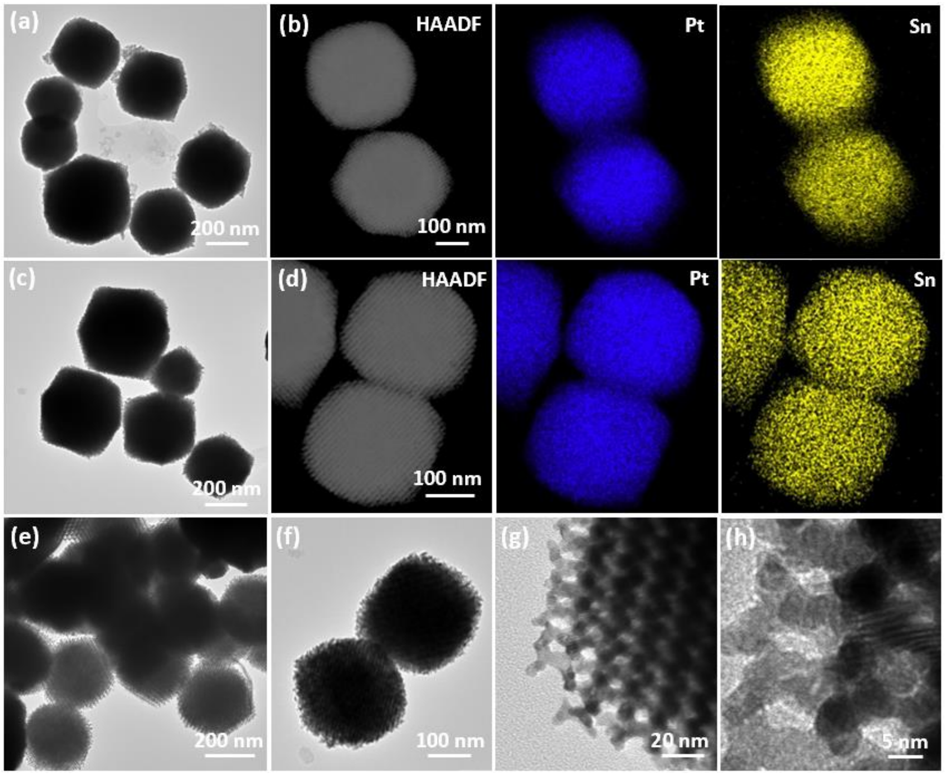

Figure S13. STEM/TEM and STEM EDX images of (a,b) *meso*-*i*-Pt_1Sn_1, (c,d) *meso*-*i*-Pt_3Sn_1, and (e-h) *meso*-Pt nanoparticles after catalysis. All the nanoparticles retained their structure/morphology and composition, indicating a good catalytic stability.

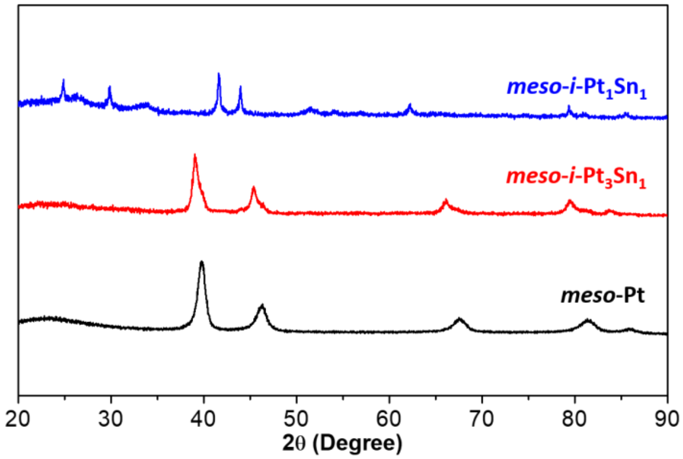

Figure S14. PXRD patterns of *meso*-Pt, *meso*-*i*-Pt_3Sn_1, and *meso*-*i*-Pt_1Sn_1 nanoparticles after catalytic stability tests. All samples retained PXRD peaks well, indicating they are chemically stable for catalysis.

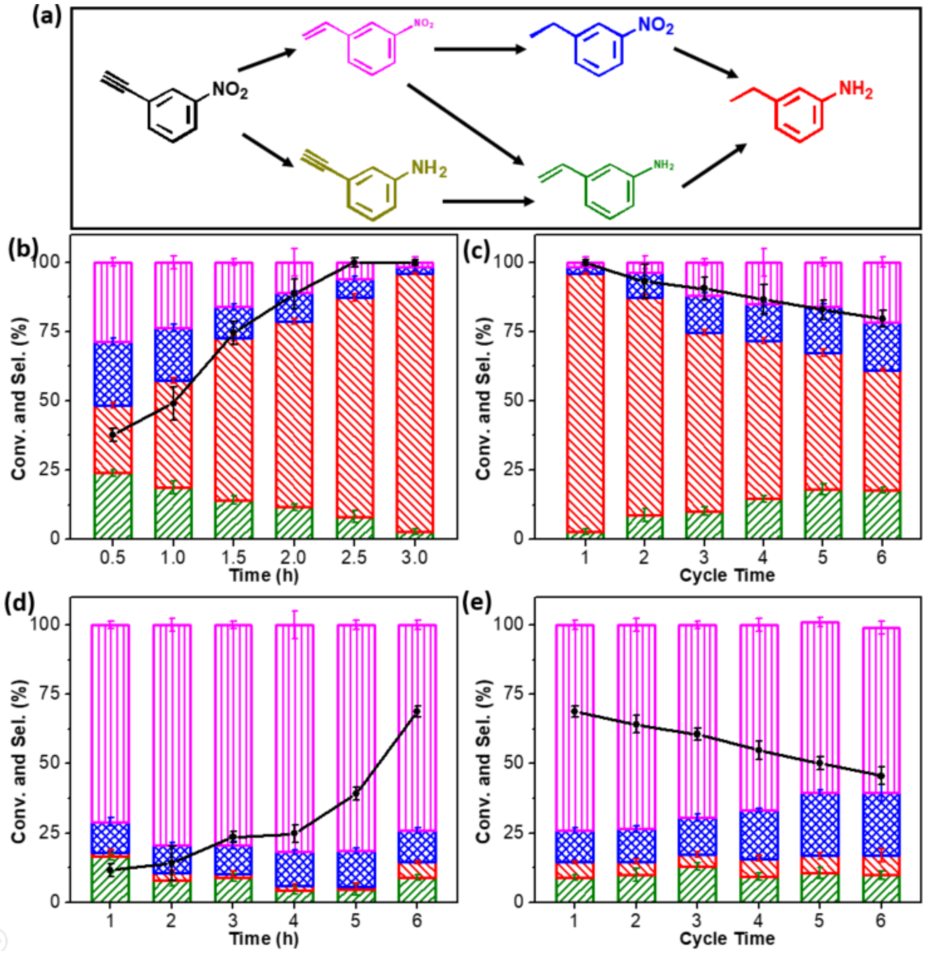

Figure S15. Catalytic performance of commercial Pt/C and *i*-Pt_1Sn_1@KIT-6. (a) Hydrogenation routes and possible products of 3-NPA. (b) 3-NPA conversion and product selectivity and (c) catalytic cycling stability over commercial Pt/C. (d) 3-NPA conversion and product selectivity and (e) catalytic cycling stability over *i*-Pt_1Sn_1@KIT-6.

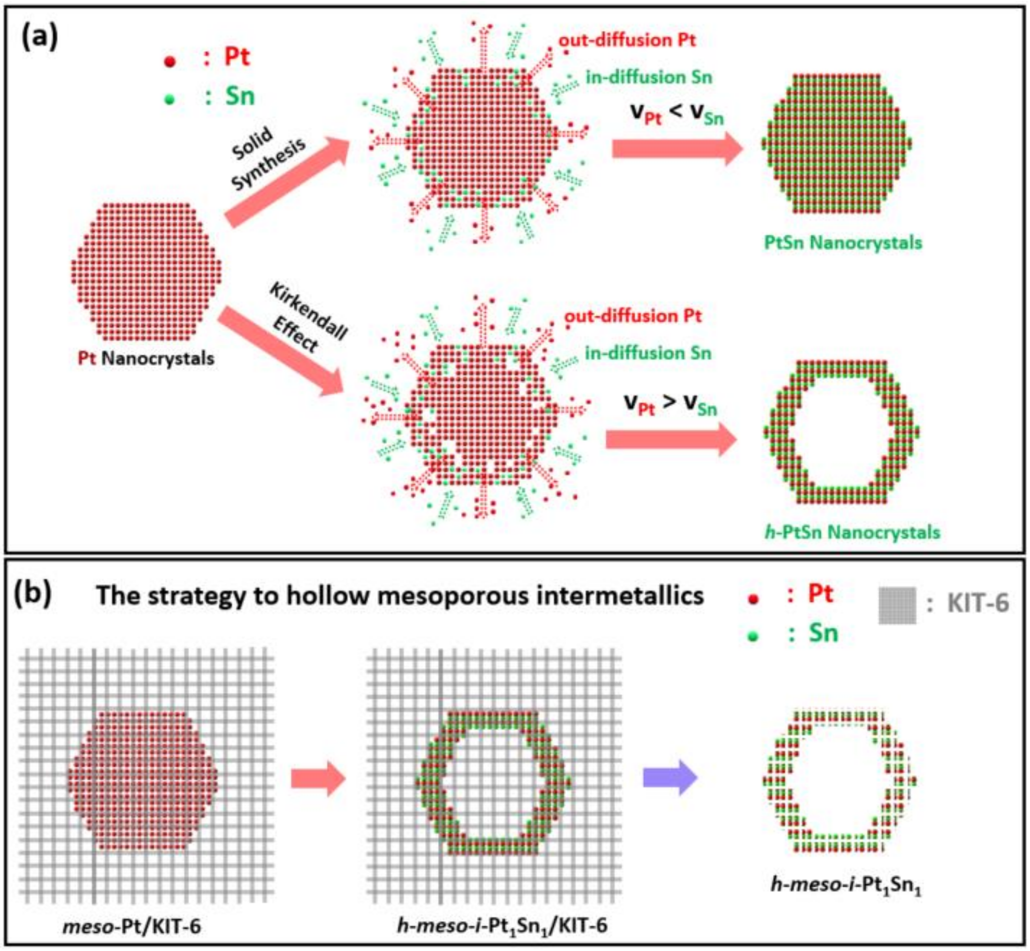

Figure S16. Synthesis of ordered *h*-*meso*-*i*-Pt_1Sn_1 nanoparticles *via* a Kirkendall-effect-directed concurrent template strategy. (a) Comparisons of traditional alloy synthesis of solid PtSn nanocrystals and Kirkendall-effect-engaged synthesis of hollow PtSn (*h*-PtSn) nanocrystals. (b) Our strategy for synthesizing *h*-*meso*-*i*-PtSn nanocrystals.

Notes for **Figure S16**: When the rate of diffusion of the *meso*-Pt nanocrystals is faster than that of recrystallization of intermetallic PtSn, the *meso*-Pt core would migrate out and produce new nanoparticle morphology. This is the well-known Kirkendall effect. Because of the nanoconfinement effect of mesoporous KIT-6 framework, the Kirkendall effect would result in the formation of hollow mesoporous intermetallic nanoparticles with ordered mesostructure and uniform interior cavity.

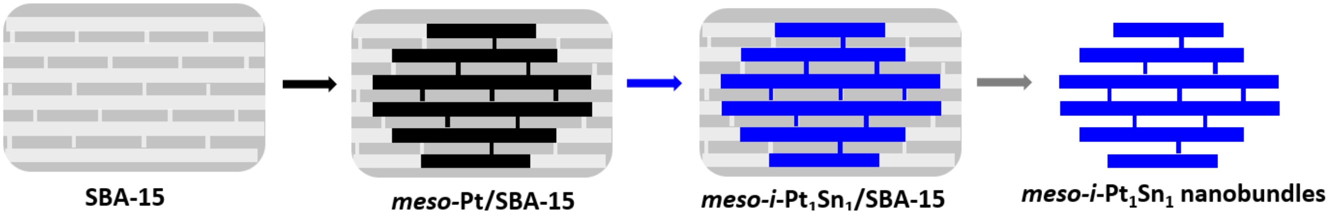

Figure S17. A schema illustrating the synthesis of ordered *meso*-*i*-Pt_1Sn_1 nanobundles *via* a concurrent template (*meso*-Pt/SBA-15) strategy. The *meso*-Pt/SBA-15 intermediate directs the synthesis of mesoporous nanobundles, since SBA-15 has a hexagonal mesostructure.

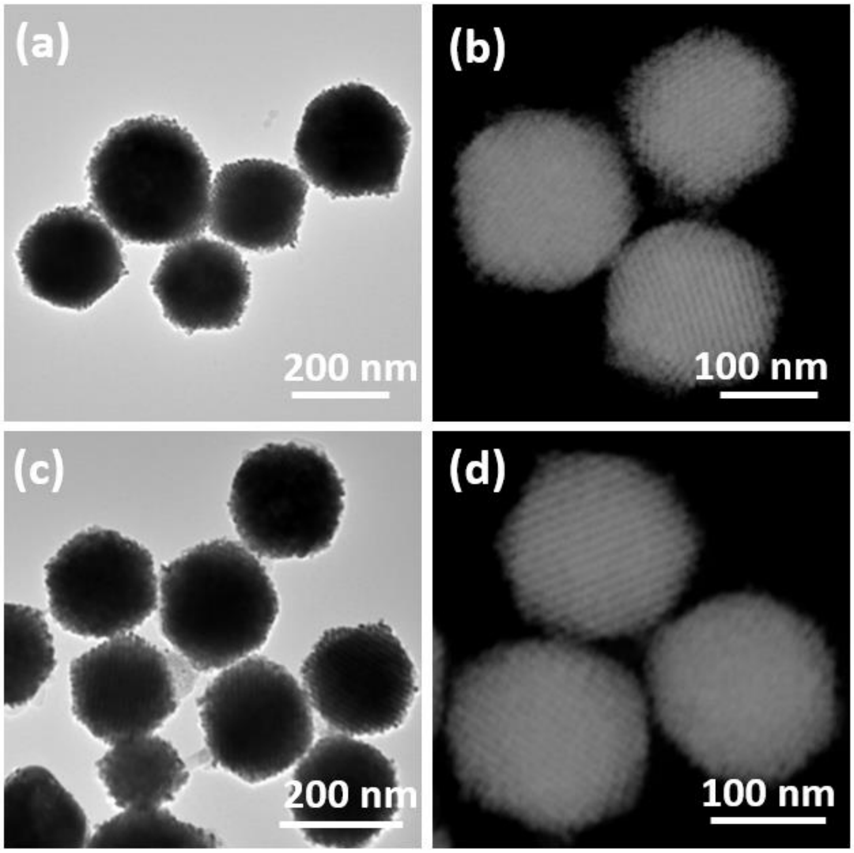

Figure S18. TEM and STEM images of (a,b) *meso*-*i*-Pt_1Cd_1 and (c,d) *meso*-*i*-Pt_1Zn_1 nanoparticles, indicating macroscopically rhombic dodecahedral morphology and mesoscopically ordered $Ia\bar{3}d$ structure. Both nanoparticles have a similar morphology and mesostructure to *meso*-*i*-Pt_1Sn_1, indicating the universality of the concurrent template strategy in synthesizing ordered mesoporous intermetallic nanoparticles.

Table S1. Pt/Sn ratios during the synthesis of *meso*-*i*-Pt_3Sn_1 and *meso*-*i*-Pt_1Sn_1 nanoparticles.

	Pt/Sn Ratio				
**To Synthesize *meso*-*i*-Pt_3Sn_1**	**KIT-6**	***meso*-Pt/KIT-6**	***meso*-Pt/KIT-6 + $SnCl_2$**	***meso*-*i*-Pt_3Sn_1/KIT-6 + $SnCl_2$**	***meso*-*i*-Pt_3Sn_1**
	**0:0**	**100:0**	**72:28**	**74:26**	**75:25**
**To Synthesize *meso*-*i*-Pt_1Sn_1**	**KIT-6**	***meso*-Pt/KIT-6**	***meso*-Pt/KIT-6 + $SnCl_2$**	***meso*-*i*-Pt_1Sn_1/KIT-6 + $SnCl_2$**	***meso*-*i*-Pt_1Sn_1**
	**0:0**	**100:0**	**40:60**	**48:52**	**50:50**
